# A Case of Arterial and Venous Tear during Single Lead Extraction

**DOI:** 10.1155/2016/3836754

**Published:** 2016-04-18

**Authors:** Michael S. Green, Daniel Wu, Vishal Patel, Rayhan Tariq

**Affiliations:** Department of Anesthesiology and Perioperative Medicine, Drexel University College of Medicine and Hahnemann University Hospital, Philadelphia, PA 19102, USA

## Abstract

Transcutaneous lead extraction can be associated with significant morbidity and mortality. The risk of causing concomitant arterial and venous injury is rare. We report a case of marginal artery rupture with coronary sinus rupture after a CS lead extraction. A 71-year-old male was admitted for extraction of a 6-year-old implantable cardioverter-defibrillator lead due to fracture from insulation break. During the lead extraction, blood pressure fell precipitously and echocardiographic findings were consistent with pericardial effusion. After unsuccessful pericardiocentesis, open chest sternotomy and evacuation of hematoma was performed. Subsequent surgical repair of several injuries was completed including the distal coronary sinus, a large degloving injury of posterior portion of the heart, and first obtuse marginal branch bleed. This case demonstrates that when performing transcutaneous lead extraction (TLE) with laser sheath, a degloving injury can cause arterial rupture with concomitant coronary sinus injury. A multidisciplinary team-based approach can ensure patient safety.* Learning Objective.* Implantable cardioverter-defibrillator leads will falter over time. With the advancement of new technology for extraction more frequent and serious complications will occur. Active fixation CS leads present unique challenges. In the presence of hemodynamic changes during extraction the occurrence of both an arterial and venous injury must be considered.

## 1. Introduction

The use of implantable cardioverter-defibrillator (ICD) or pacemakers has increased significantly over the past 2 decades. These mechanical devices are subject to deterioration and malfunction. Although any component of the implantation can malfunction, the lead is the most susceptible [1]. The estimated lead survival rate at 5 years after implantation is 85% and at 8 years is 60%, reaching down to 20% at 10 years [2]. At such time, leads need to be removed through lead extraction. Leads can be extracted via a transcutaneous approach. Transcutaneous lead extraction (TLE) can be associated with significant morbidity and mortality. Reported major complication and mortality rates with TLE vary widely across studies. In one large study the rate of major complications in TLE was found to be 1.4% [3]. These complications range from cardiac tear, vascular avulsion, pulmonary embolism, and anesthesia related complications. These complications can require blood transfusion or surgical intervention. The leads are designed so they are advanced and fixed in the coronary venous system around the heart, so there is an unavoidable risk of causing venous injury during the traction and dislodging. However, the risk of causing arterial injury during lead extraction is rare. We report a case of marginal artery rupture along with coronary sinus rupture after an ICD lead extraction. Truly a rare occurrence was unreported in the literature.

## 2. Case Report

A 71-year-old male with a history of chronically implanted ICD (6 years) was initially admitted to electrophysiology (EP) suite for an ICD lead extraction and replacement upgrade of the system to a biventricular functional system. During an ICD pulse generator replacement (*Medtronic Viva XT CRT-D, model DTBA1D1*) 2 months earlier the coronary sinus lead was noted to have a fracture due to insulation break so it was cut and capped at that time. The model of this particular implanted Left Ventricular Coronary sinus lead was* Medtronic StarFix unipolar active fixation, model 4195* (implant date 6 years ago). Patient had history significant for class III congestive heart failure with underlying LBBB.

Cardiac fluoroscopy was performed to demonstrate normal position of the ICD pulse generator in the left subclavian area. The right atrial and ventricular leads tracked normally while the coronary sinus lead was noted to be fractured within the pocket with the lead remnant in the ICD pocket. Cardiac silhouette motion was normal and no evidence of pleural effusion was noted. Local anesthesia was infiltrated to the right and left groin. An 18 g arterial line was placed in the left femoral artery with good waveform. #7f and #9f venous lines were placed in the right femoral vein. Through the #9f, an intracardiac echocardiographic (ICE) probe was advanced to the level of the right atrium and ventricle. No evidence of vegetation or pericardial effusion was noted. The left subclavian area was prepped and draped. Local anesthesia was administered to the left anterior chest wall prior to opening the pocket. The pulse generator was removed from the pocket and leads were dissected to the level of the anchoring sleeves. There was a cap present over the abandoned lead remnant and this cap was removed. Tie-down sutures were removed. Leads were dissected to the level of the anchoring sleeves to free them up from posterior scar tissue. The anchoring sleeve of the coronary sinus lead was removed and gentle traction initiated; however, it was heavily scarred into position at the distal branch of the lateral branch of the coronary sinus. All 4 splines were deployed. The head of the lead was cut off and a Liberator locking stylet was advanced and locked at 1 cm from the tip of the lead. With continuous traction, a 12-French laser sheath was advanced over the lead to break up heavy fibrosis near the left brachiocephalic vein and down to the superior vena cava. The lead became dislodged situating itself at the proximal area of the coronary sinus. Continuous gentle traction of this area and laser at the ostium of the coronary sinus lead dislodged the lead in its entirety from the coronary sinus and it was removed without difficulty. There was a substantial amount of scarring at the level of the coronary sinus insertion site and around the splines before they became dislodged.

At this time, the patient's blood pressure became labile. Cardiac silhouette motion was decreased and ICE probe showed pericardial effusion consistent with pericardial tamponade. A subxiphoid pericardiocentesis needle was inserted and 60 cc of blood was aspirated. The patient's blood pressure responded and, however, subsequently started to decrease again. Cardiothoracic surgery was called to the EP suite, and patient was intubated and requiring massive hemodynamic support, which deteriorated into cardiac arrest requiring cardiopulmonary resuscitation. While in the EP suite, open chest sternotomy and evacuation of hematoma from the pericardium was performed. Large amount of clot was removed from behind the heart and hemodynamics stabilized despite continued bleeding. Patient was transferred to the operating room.

Intraoperatively, the patient was immediately placed on cardiopulmonary bypass to explore cardiac structures and injury. Several injuries were noted including a large amount of bleeding from the posterior portion of the heart due to total disruption of the distal coronary sinus and large degloving injury of posterior portion of the heart with large first obtuse marginal branch bleed. In addition, the innominate vein had a large hematoma overlying a tear where it crossed the aorta. At least 2 puncture injuries to the heart were noted likely associated with pericardiocentesis.

After the patient's status stabilized, electrophysiology recommended replacement of biventricular ICD pulse generator to his remaining right atrial and ventricular leads without any further manipulation of the leads with intention to eventually upgrade to a biventricular device. The procedure was successfully completed.

## 3. Discussion

Most pacemaker leads implanted within a year can be removed without the use of any specialized equipment, termed “lead explant” [4]. But as the duration of the leads increases, fibrosis will form around the leads and adhere them to the vessel walls. thus requiring specialized equipment in a procedure named “lead extraction” [4]. Therefore, in patients with a chronically implanted lead, more fibrosis will form around the lead, causing the extraction procedure to be more difficult and prone to complication. The most common reasons for lead extraction are device infection and lead conduction failure. A multidisciplinary team-based approach involving a cardiothoracic surgeon, anesthesia support, access to fluoroscopy and echocardiography, and nursing support is critical in order to ensure safe outcome [4]. Operator experience is also an important component in reducing complications. A single center's experience using a multidisciplinary approach suggests that there is a learning curve to lead extraction procedure as well as the preparation for potentially unavoidable complications [4]. Although infrequent, most of the complications attempting the extraction involved a laser sheath technique, leading to SVC tear or cardiac tamponade [5, 6]. A trend toward more complication was noted in procedures associated with a laser sheath (3.4% versus 0.8%) [6]. With an increased amount of fibrosis around the leads, a laser approach is more desired to remove the fibrotic tissue around the lead for an easier extraction. The increased incidence rate of complications using the laser sheath could be due to decreased cardiac wall stiffness after lasing with a larger sized sheath (12f–16f) [7]. A decreased wall stiffness could cause the pericardial tissue to tear more easily under the stress of extraction. In this case, avulsion of the lead caused a large degloving injury during which left marginal artery was ruptured. Left marginal artery (or obtuse marginal artery) is a branch of the circumflex artery, traveling along the left margin of heart towards the apex of the heart. Active fixation CS lead such as the Medtronic StarFix CS lead used in our case presents additional procedural complexity compared to the use of passive fixation CS lead. It almost always required the use of excimer laser sheath compared and reimplantation proved to be impossible in the same venous branch [8].

Active fixations CS leads were introduced to reduce the rate of lead dislodgment, but the active fixation mechanism can present with added complications should these leads necessitate extraction. Although limited knowledge base in the literature has reported acceptable success and morbidity in extraction of active CS leads [9], there should always be an anticipation and preparation for any potential complication. Our case demonstrated that when performing a transvenous lead extraction of an active fixation CS lead, a degloving injury can cause arterial rupture and venous rupture. A multidisciplinary team-based approach can ensure patient safety when faced with complications such as a coronary sinus and arterial rupture.
